# Stimulation of non-motor subthalamic nucleus impairs selective response inhibition via prefrontal connectivity

**DOI:** 10.1093/braincomms/fcad121

**Published:** 2023-04-13

**Authors:** Josefine Waldthaler, Alexander Sperlich, Charlotte Stüssel, Kenan Steidel, Lars Timmermann, David J Pedrosa

**Affiliations:** Department of Neurology, University Hospital Gießen and Marburg, 35033 Marburg, Germany; Center for Mind, Brain and Behavior (CMBB), Philipps-University Marburg and Justus-Liebig-University Giessen, 35033 Marburg, Germany; Department of Clinical Neuroscience, Karolinska Institute Stockholm, 17165 Solna, Sweden; Department of Neurology, University Hospital Gießen and Marburg, 35033 Marburg, Germany; Department of Neurology, University Hospital Gießen and Marburg, 35033 Marburg, Germany; Department of Neurology, University Hospital Gießen and Marburg, 35033 Marburg, Germany; Department of Neurology, University Hospital Gießen and Marburg, 35033 Marburg, Germany; Center for Mind, Brain and Behavior (CMBB), Philipps-University Marburg and Justus-Liebig-University Giessen, 35033 Marburg, Germany; Department of Neurology, University Hospital Gießen and Marburg, 35033 Marburg, Germany; Center for Mind, Brain and Behavior (CMBB), Philipps-University Marburg and Justus-Liebig-University Giessen, 35033 Marburg, Germany

**Keywords:** deep brain stimulation, Parkinson’s disease, eye tracking, connectome, impulsivity

## Abstract

Given the inconsistent results in the past, there is an ongoing debate whether and how deep brain stimulation in the subthalamic nucleus modifies cognitive control processes like response inhibition in persons with Parkinson’s disease. In this study, we examined how the location of the stimulation volume within the subthalamic nucleus affects the performance in an antisaccade task but also how its structural connectivity is related to response inhibition. Antisaccade error rates and latencies were collected in 14 participants on and off deep brain stimulation in a randomized order. Stimulation volumes were computed based on patient-specific lead localizations using preoperative MRI and postoperative CT scans. Structural connectivity of the stimulation volumes with pre-defined cortical oculomotor control regions as well as whole-brain connectivity was estimated using a normative connectome. We showed that the detrimental effect of deep brain stimulation on response inhibition, measured as antisaccade error rate, depended upon the magnitude of the intersection of volumes of activated tissue with the non-motor subregion of the subthalamic nucleus and on its structural connectivity with regions of the prefrontal oculomotor network including bilateral frontal eye fields and right anterior cingulate cortex. Our results corroborate previous recommendations for avoidance of stimulation in the ventromedial non-motor subregion of the subthalamic nucleus which connects to the prefrontal cortex to prevent stimulation-induced impulsivity. Furthermore, antisaccades were initiated faster with deep brain stimulation when the stimulation volume was connected to fibres passing the subthalamic nucleus laterally and projecting onto the prefrontal cortex, indicating that improvement of voluntary saccade generation with deep brain stimulation may be an off-target effect driven by stimulation of corticotectal fibres directly projecting from the frontal and supplementary eye fields onto brainstem gaze control areas. Taken together, these findings could help implement individualized circuit-based deep brain stimulation strategies that avoid impulsive side effects while improving voluntary oculomotor control.

## Teaser

Waldthaler *et al*. investigated how location and structural connectivity of the deep brain stimulation volume relate to response inhibition capacity measured in the antisaccade task, finding that stimulation-induced deterioration of response inhibition depends upon the magnitude of stimulation in the non-motor subregion of the subthalamic nucleus and on its connectivity with the prefrontal cortex.

## Graphical Abstract

**Graphical Abstract:**
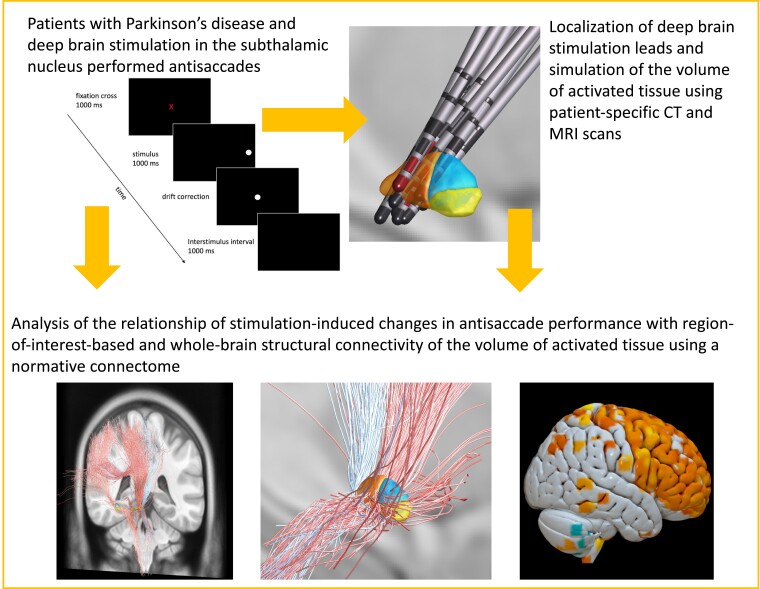

## Introduction

There is no debate about the effectiveness of deep brain stimulation (DBS) in the subthalamic nucleus (STN) for motor symptoms of Parkinson’s disease. Its effects on cognitive and oculomotor functions, on the other hand, remain less clear. Given that the STN has been deemed pivotal for cognitive control processes like response inhibition (i.e. the ability to stop or withhold a reflexive prepotent response), alterations thereof following DBS seem plausible. The results in the literature are yet heterogeneous with studies reporting STN-DBS impairing^1-3^ or improving response inhibition,^4,5^ or leaving it unaltered.^6-8^ A recent meta-analysis did not confirm an overall detrimental effect of STN-DBS on inhibitory control in Parkinson’s disease,^9^ so that one may infer differences in task design (global versus selective response inhibition) conditioning these inconsistent findings.^10^ Furthermore, analogous to motor symptoms, there could be a differential effect of DBS on cognitive symptoms depending on the extent of the volume of activated tissue (VAT) within the STN and therefore the activated circuitry.^11,12^

Best motor symptom control results from stimulation in the dorsolateral ‘motor’ subregion of the STN which receives projections predominantly from the primary and supplementary motor cortices.^13-15^ However, despite optimal lead placement and refined DBS programming, VAT may exert effects in ventral and medial portions of the STN and to surrounding structures and fibre tracts, alike. In contrast to the motor subregion, the adjacent ventromedial portions of the STN receive converging cortical projections from prefrontal areas including the pre-supplementary motor area (pre-SMA), the frontal eye field (FEF), dorsolateral prefrontal cortex (DLPFC) and anterior cingulate cortex (ACC);^13^ all regions forming an interconnected network involved in response inhibition.^16,17^ The exact lead location and VAT within the STN may thus crucially determine effects of DBS on cognitive performance. For instance, DBS of the ventromedial ‘associative’ subregion of the STN has been related to errors of inhibition, and more broadly with clinically relevant neuropsychiatric symptoms and with DBS-associated cognitive decline.^18,19^

**Figure 1:**
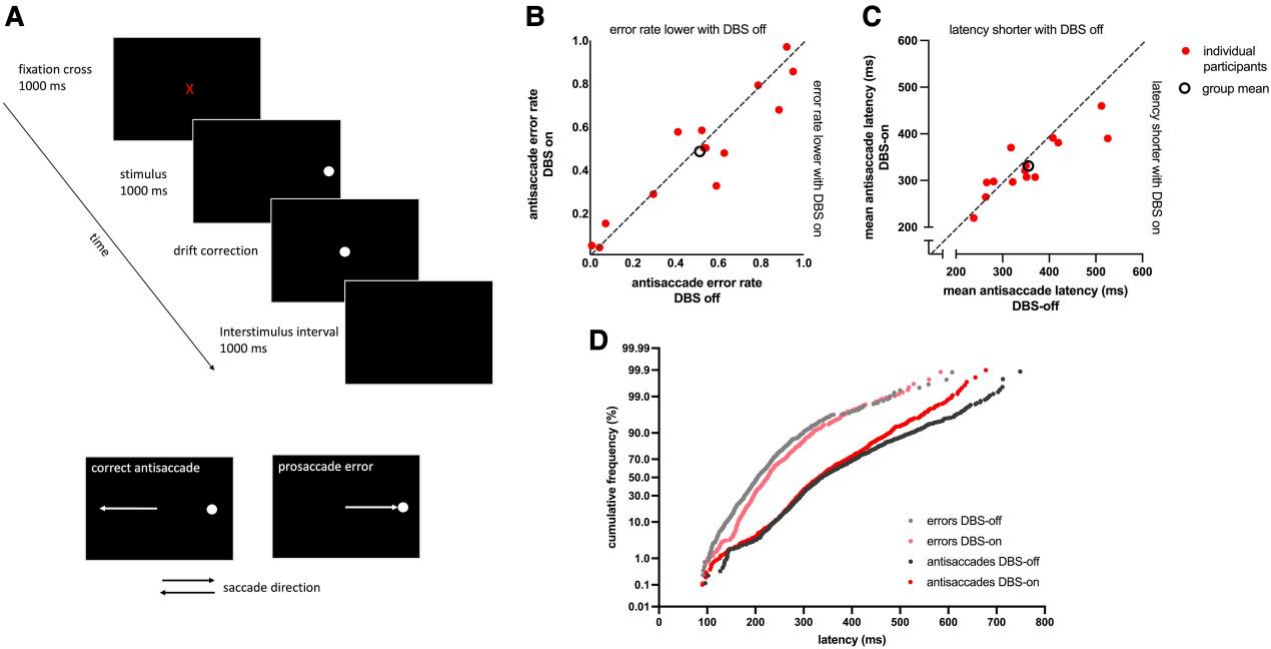
**Results of the antisaccade task.** (**A**) Depiction of the antisaccade task. (**B–C**) Scatter plots comparing the mean of antisaccade error rate (**B**) and latency (**C**) in the DBS-off condition (*x*-axis) and DBS-on conditions (*y*-axis) within participants. Individual values for each participant are displayed as dots; the black circle represents the group mean. The unity line is shown as a dark grey dashed line. (**D**) Cumulative frequency distributions of latencies with probit scaled *y*-axis pooled across all participants. Each dot represents a single trial. Data from a total of 858 and 992 correct antisaccade trials, respectively 811 and 832 erroneous trials, were included for the DBS-off and DBS-on condition respectively. Correct antisaccades displayed in intense colours and errors in faint colours. DBS-on condition in red, DBS-off condition in dark grey.

The aim of this study was therefore to examine how the position of the VAT in the STN as well as its structural connectivity relates to the individual effect of STN-DBS on response inhibition in Parkinson’s disease. To control for DBS-induced improvement in manual responses, we used the antisaccade paradigm, an established response inhibition task in the oculomotor domain. Antisaccades require the selective suppression of a reflexive saccade in the direction of a visual stimulus (the *pro*saccade) and the execution of a voluntary saccade in the opposite direction instead (the *anti*saccade) (Fig. 1A).^20^ The rate of directional errors towards the visual stimulus serves as proxy for response inhibition capacity, while the latency (i.e. the duration between onset of the visual target and the onset of the saccade) additionally measures the efficiency with which the voluntary action is selected and initiated by the oculomotor control network.

While Parkinson’s disease-patients tend to exceed healthy individuals in antisaccade error rates and latencies,^21-23^ effects of STN-DBS on antisaccade performance are not fully understood. A recent meta-analysis found an overall decreasing effect of STN-DBS on antisaccade latency but no consistent effect on the rate of directional errors.^24^ We hypothesized that inconsistent results of previous studies may be related to variability in the exact location of the DBS lead and the VAT within the STN and its surroundings.

## Materials and methods

### Participants

The study was approved by the Ethical Board of the University Hospital Marburg (reference number 119/19) and followed the Declaration of Helsinki. All participants were recruited from the Movement Disorders Outpatient Clinic of the Department of Neurology at the University Hospital Marburg and gave written informed consent before participation.

A total of 19 subsequent participants with Parkinson’s disease diagnosed according to the clinical diagnostic criteria of the Movement Disorders Society^25^ treated with STN-DBS participated in this study. Exclusion criteria were (i) mild cognitive impairment or dementia according to the respective MDS task force criteria level 1 and measured with the Montreal Cognitive Assessment (< 24 points), (ii) signs of clinically relevant depression (Beck Depression Inventory > 14 points), (iii) history of any disorder of the central nervous system other than Parkinson’s disease, (iv) any concurrent conditions that made eye-tracking impossible (for example, disorders of the eyes or visual system with reduced visual acuity, severe camptocormia and other orthopaedic disorders impairing ability to sit for longer periods) and (v) intake of any medications that may influence eye movements (for example, benzodiazepines).

All participants were in off-medication state after overnight withdrawal of all dopaminergic medication for at least 12 h prior to study assessments.

Five of 19 participants asked for premature discontinuation of the study protocol due to tiredness, discomfort or unbearable motor symptoms in the DBS-off state. Fourteen participants completed at least one block of antisaccade recordings in both conditions and were included in the final analysis.

### DBS programming

All patients were implanted bilaterally with leads targeting the sensorimotor part of STN (Vercise Cartesia™ Directional Lead, Boston Scientific Neuromodulation Corporation, Valencia, CA91355, USA). These DBS leads consist of eight contacts configured into two ring contacts at the proximal and distal pole and two three-segment contacts in-between, enabling directionally shaped VAT.^26^ The minimum time span between DBS surgery and study inclusion was 3 months to avoid any impact of lesion effects.

All participants performed the task twice: with DBS switched off, and in their chronic DBS program that achieved optimal clinical response (Supplementary Table 1). The order of conditions was randomized to avoid bias due to expectations, learning effects or tiredness. The participants were not informed about what changes to their DBS settings were made. The wash-out period between conditions was at least 10 min.

### Eye-tracking procedure and analysis

The experiment was programmed in MATLAB 2020b (The Mathworks Inc., Massachusetts, USA) using the psychophysics toolbox (www.psychtoolbox.org)^27^ and an infrared video-based eye-tracker (EyeLink 1000 Plus, SR Research, Ontario, Canada) recorded positions of both eyes. Three blocks of 50 horizontal antisaccades in each were presented per condition, resulting in *N* = 150 trials per condition. Participants were instructed to look at the exact opposite direction of the lateral target stimulus located either 10° left or right from an initial central fixation cue as fast and precisely as possible (Fig. 1A).

After segmentation of the raw pupil position data into visual events (saccades, fixations and blinks) using a parsing system incorporated in the EyeLink 1000 software, the event data set was imported into the statistical computing program R^28^ using the Eyelinker package for further analysis (cf. Supplementary material 1 for detailed description of the eye-tracking procedure and analysis).

Antisaccade errors were defined as a saccade towards the lateral stimulus. Saccade latency was defined as the time from stimulus onset to the start of the first saccade. Saccades occurring with very short latency < 90 ms were considered anticipatory and removed from further analysis. The following oculomotor outcome measures were extracted: (i) error rate (proportion of erroneous trials with latency > 89 ms to all valid trials), and (ii) mean latency of correctly executed antisaccades.

### Visualization of DBS leads and VAT

Image processing was performed using the pipeline provided in the Lead-DBS software (https://www.lead-dbs.org).^29,30^ In brief, pre-operative T2-weighted MRI scans were linearly co-registered to T1-weighted images using SPM12 (http://www.fil.ion.ucl.ac.uk/spm/software/), and subsequently co-registered to postoperative CT scans using a two-stage linear registration as implemented in Advanced Normalization Tools.^31^ The co-registered acquisitions were then spatially normalized into ICBM 2009b NLIN asymmetric standard space^32^ using Advanced Normalization Tools.^33^ The localizations of DBS leads were corrected for brainshift by applying a refined affine transform calculated between pre- and postoperative acquisitions restricted to a subcortical area of interest. After pre-localization of the DBS electrodes using the PaCER algorithm,^34^ their location was manually corrected based on postoperative CT scans using the respective tool as implemented in Lead-DBS software. Consecutively, the orientation of the directional DBS leads was determined using the default algorithm in the package.^35,36^ Electrode locations and active contacts were visualized with Lead-Group.^37^

Based on the individual DBS settings of each patient, VAT were estimated using a finite element method with an electric field threshold of 0.2 V/mm.^15,29^ This model estimated the E-field (i.e. the gradient distribution of the electrical charge in space measured in V/mm) on a tetrahedral mesh that differentiated four compartments (grey and white matter, electrode contacts and insulation) whereby the grey matter structures including a parcellation of the STN subregions were defined using the DISTAL Minimal Atlas.^38^ Consecutively, patient-specific VAT were used to calculate their intersection with the motor and non-motor (associative and limbic area combined) subregions of the STN in proportion to total VAT volumes. Finally, these normalized intersection values were averaged between right and left hemisphere for each participant and extracted for further statistical analysis.

### Region-of-interest based structural connectivity

The supplementary eye field (SEF), FEF, DLPFC and ACC were selected as *a priori* regions of interest (ROI) based on their known activation during antisaccades and strong association with antisaccade latency and error rate.^39-42^ The ROI were defined using the Brainnetome atlas^43^ as previously used in^44^ (Supplementary Table 2). Structural connectivity between VAT and ROI was calculated using a publicly available normative connectome derived from diffusion-weighted MRI of 85 individuals with Parkinson’s disease recruited for the Parkinson’s Progression Markers Initiative (PPMI, www.ppmi-info.org).^38,45^ Regions of interest and patient-specific VAT were projected onto the voxelized volume of the connectome in standard space in 1 mm isotropic resolution. Structural connectivity seeding from the VAT and projecting onto voxels of the respective ROI was estimated for each participant as fibre counts, i.e. numbers of streamlines seeding from VAT.

### Whole-brain structural connectivity

To test whether the brain-wide connectivity of the VAT predicted DBS effects on antisaccade measures, we used patient-specific VAT as seed regions to all fibre tracts of the normative connectome. Then, VAT were grouped depending on whether they touched each of the fibres of the connectome or not. The change scores of antisaccade latency, respectively error rate, were compared between connected and unconnected VAT for all fibres using mass *t*-tests resulting in a *t*-score for each fibre. Using this approach as previously described by Li and colleagues,^46^ a high *t*-score reflects that a specific fibre is highly explanatory for the DBS effect on the respective oculomotor measure. Only fibres within the 20% highest *t*-scores, i.e. those with the highest discriminative power, were kept for further statistical analysis.

### Statistical analysis

For all analyses, statistical inference was ascertained in R and statistical significance was asserted at α = 0.05. Mean antisaccade latency and error rate were compared on a group level between DBS-off and DBS-on conditions using paired *t*-tests. Absolute change scores (ΔDBS-on = DBS-off) were calculated for correlation analyses with imaging data so that a negative change score indicates a DBS-induced decrease from DBS-off state values.

Since DBS-induced changes in antisaccade latency and error rate were not expected to be normally distributed, change scores of the oculomotor outcomes were Spearman rank-correlated with the VAT-STN intersections with the motor and non-motor subregions and with fibre counts connecting the VAT to the pre-defined ROI.

Group-level cross-validation of the derived whole-brain fibre connectivity profiles was performed using a leave-one-out approach.^47^ Each patient was excluded once to subsequently re-calculate discriminative fibres based on the fibre connectivity profile generated using the remaining 13 participants. A spatial correlation coefficient was calculated comparing the similarity between the profiles of the single individual with the profile of the remaining cohort. This spatial correlation coefficient was entered into a linear regression model trained on the remaining cohort to predict the individual DBS effects on oculomotor measures of the excluded patient.

For visualization purposes, the strength of structural connectivity was additionally calculated in a voxel-wise manner. Here, the fibre density connecting the VAT with each voxel of the connectome was Spearman rank-correlated with the change in antisaccade latency and error rate across participants.^48^ The resulting connectivity maps (‘R-maps’) represent the Spearman’s correlation coefficients in each voxel color-coded for positive and negative correlations.

### Data availability

The code for pre-processing of the imaging data, lead localization, VAT generation and connectivity analysis is freely accessible through the Lead-DBS software (www.lead-dbs.org).^29^ All newly generated custom-written code for the task (Matlab) and analysis pipelines (R) is available on the following GitHub page: https://github.com/JoWld/PD_DBS_Antisac. Participants’ individual data sets are available upon reasonable request from the corresponding author.

## Results

### Participants’ clinical characteristics

The final analysis resulted from 14 participants (four females) aged of 57.0 ± 8 8 years and with a mean disease duration of 8.6 ± 3.3 years. Mean time between study inclusion and DBS surgery was 10.2 ± 9.1 months. All participants experienced improvement of motor symptom severity when DBS was switched on with a mean relative reduction in MDS-UPDRS III scores of 56.9 ± 21.8%.

### Behavioural results

See Table 1 for condition-averaged results.

**Table 1:** Group-averaged behavioural results of the antisaccade task without significant effects of DBS-condition (paired *t*-tests with 13 degrees of freedom)

	DBS-off	DBS-on	T statistic	*P*
Error rate, mean (sd)	0.514 (0.318)	0.486 (0.290)	0.888	0.4
Antisaccade latency (ms), mean (sd)	355.1 (87.2)	331.1 (61.6)	1.986	0.1

Individual DBS-induced change in antisaccade error rate differed considerably between participants (Fig. 1B). Paired *t*-test revealed no evidence for a difference between conditions (DBS-off/DBS-on, *t*(13) = 0.888, *P* = 0.391) with a mean decrease in antisaccade error rate by −0.027 ± 0.116 (*95%-CI* = [−0.094, 0.039]) when DBS was switched on.

Paired *t*-test further revealed no significant effect of condition on antisaccade latency [*t*(13) = 1.986, *P* = 0.069] with a mean decrease by 24.0 ± 45.2 ms [*95%-CI* = (−2.1, 50.1)] in DBS-on condition whereby the confidence interval indicated a trend towards a DBS-induced latency reduction (Fig. 1C). To further visualize the distribution of latencies across trials, the relative latency distributions of all trials are shown color-coded for condition in Fig. 1D.

Explorative correlations between change scores of oculomotor outcomes and clinical variables (age, disease duration, MoCA score and MDS-UPDRS III change score) were not significant (Supplementary Table 3).

### Localization of DBS leads and VAT

**Figure 2:**
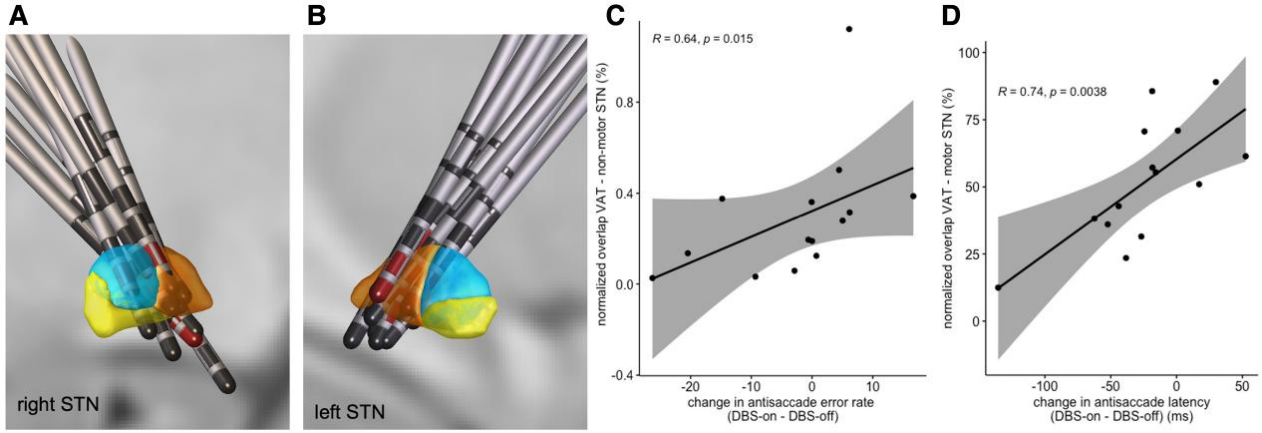
**Lead positions in standard space (ICBM 2009b NLIN asymmetric) in the subthalamic nucleus (STN) in anterior view.** Right STN in **A** and left STN in **B**. Motor subregion of the STN in orange, non-motor subregion in cyan (associative) and yellow (limbic) taken from the DISTAL atlas. Active contacts are shown in red. (**C–D**) Significant Spearman correlations of VAT intersections with the STN subregions and DBS-induced changes in antisaccade error rate and latency respectively. 95% confidence interval in grey.

See Fig. 2 for the location of all DBS leads in standard space. The bilateral VAT generated by all activated contacts were located to some extent within the STN. Normalized to the total volume of the respective VAT, 51.8 ± 22.6% [range (12.4, 89.0)] of the VAT were localized within the motor subregion, and 14.6 ± 14.0% [range (1.3, 56.0)] in the non-motor subregion of the STN.

### Correlation of antisaccade outcomes and VAT

The normalized VAT intersection with the non-motor subregion of the STN was positively correlated with the change score of antisaccade error rate (*ρ* = 0.644, *P* = 0.013). That means that a larger proportion of the VAT localized in the non-motor STN subregion was associated with a larger DBS-induced increase of errors (Fig. 2C, Table 2).

**Table 2:** Spearman correlations of the changes scores of oculomotor measures with the normalized VAT-STN intersections

	Normalized intersection VAT—motor STN		Normalized intersection VAT—non-motor STN	
	ρ	*P*	ρ	*P*
Change in antisaccade latency	0.736	0.003	−0.165	0.573
Change in regular error rate	−0.042	0.887	0.644	0.013

Conversely, the change in antisaccade latency was positively correlated with the VAT intersection with the motor subregion of the STN (*ρ* = 0.736, *P* = 0.003). That means that a larger proportion of the VAT located in the motor STN subregion was associated with less DBS-induced reduction in antisaccade latency (Fig. 2D).

### Structural connectivity with cortical oculomotor network

**Figure 3:**
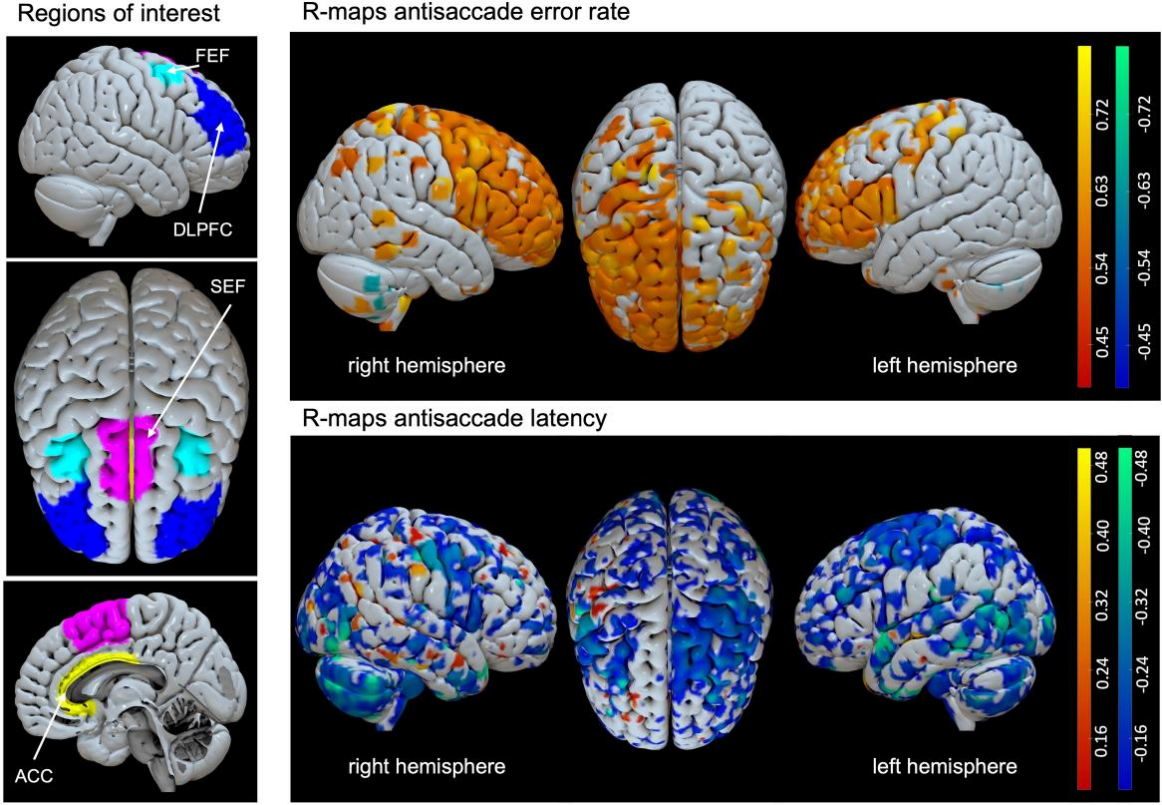
**Whole-brain structural connectivity maps seeding from bilateral VAT. Regions of interest considered in the fibre tract-based analysis (left).** Fibre density connecting the VAT with each voxel of the connectome was Spearman rank-correlated with the change score of antisaccade error rate (upper row) and antisaccade latency (lower row). Spearman’s correlation coefficients colour-coded for positive and negative correlations for each voxel of the normative connectome. In the leave-one-out validation, the DBS effect of single individuals on error rate (*R* = −0.57, *P* = 0.017) but not latency (*R* = − 0.29, *P* = 0.141) was predictable by linear models trained on the respective R maps of the remaining 13 participants. ACC, anterior cingulate cortex; DLPFC, dorsolateral prefrontal cortex; FEF, frontal eye field; SEF, supplementary eye field.

**Figure 4:**
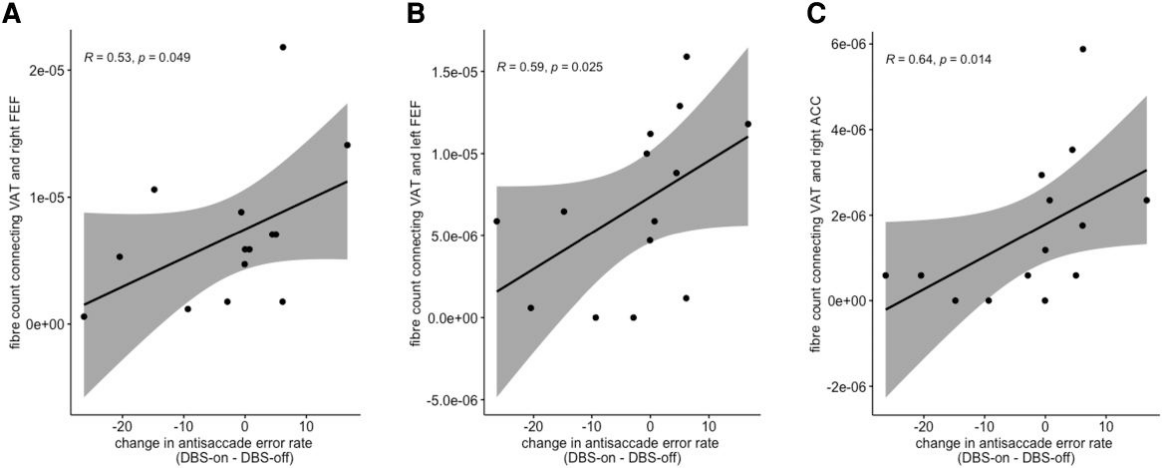
**Correlations of fibres seeding from the volume of activated tissue and projecting to regions of interest with DBS-induced changes in antisaccade error rate.** Only significant spearman correlations for fibre counts (numbers of streamlines seeding from VAT) projecting to the left frontal eye field (**A**), right frontal eye field (**B**) and right anterior cingulate cortex (**C**) are shown. 95% confidence interval in grey. ACC, anterior cingulate cortex; FEF, frontal eye field.

Considering all fibres connecting the VAT with voxels of the pre-defined ROI in the oculomotor network (Fig. 3), significant positive correlations with the change score of the antisaccade error rate were found for the right ACC (*ρ* = 0.637, *P* = 0.014), right FEF (*ρ* = 0.534, *P* = 0.025) and left FEF (*ρ* = 0.595, *P* = 0.049) (Fig. 4). That means that higher fibre counts connecting the VAT with the right ACC and bilateral FEF were associated with a larger DBS-induced increase in errors. Surprisingly, no such association was found for the DLPFC despite its known key role in the antisaccade task.^49^ However, strong correlations between voxels of the right DLPFC and the change in error rate were evident in the whole-brain connectivity map (Fig. 3).

There were no significant correlations of the change score of antisaccade latency with fibre counts connected to the FEF, SEF, DLPFC or ACC (Table 3).

**Table 3:** Spearman correlations of the changes scores of oculomotor measures with fibre counts connecting bilateral volumes of activated tissue with prefrontal regions of oculomotor control network

	Right DLPFC		Left DLPFC		Right SEF		Left SEF		Right FEF		Left FEF		Right ACC		Left ACC	
	ρ	*P*	ρ	*P*	ρ	*P*	ρ	*P*	ρ	*P*	ρ	*P*	ρ	*P*	ρ	*P*
Change in antisaccade latency	0.182	0.534	0.209	0.473	−0.086	0.771	−0.196	0.503	−0.099	0.736	−0.293	0.309	−0.036	0.903	−0.538	0.057
Change in regular error rate	0.253	0.384	0.172	0.557	**0.481**	**0.081**	0.156	0.594	**0**.**534**	**0**.**049**	**0**.**595**	**0**.**025**	**0**.**637**	**0**.**014**	0.247	0.395

Significant correlations in bold.

### Whole-brain structural connectivity

**Figure 5:**
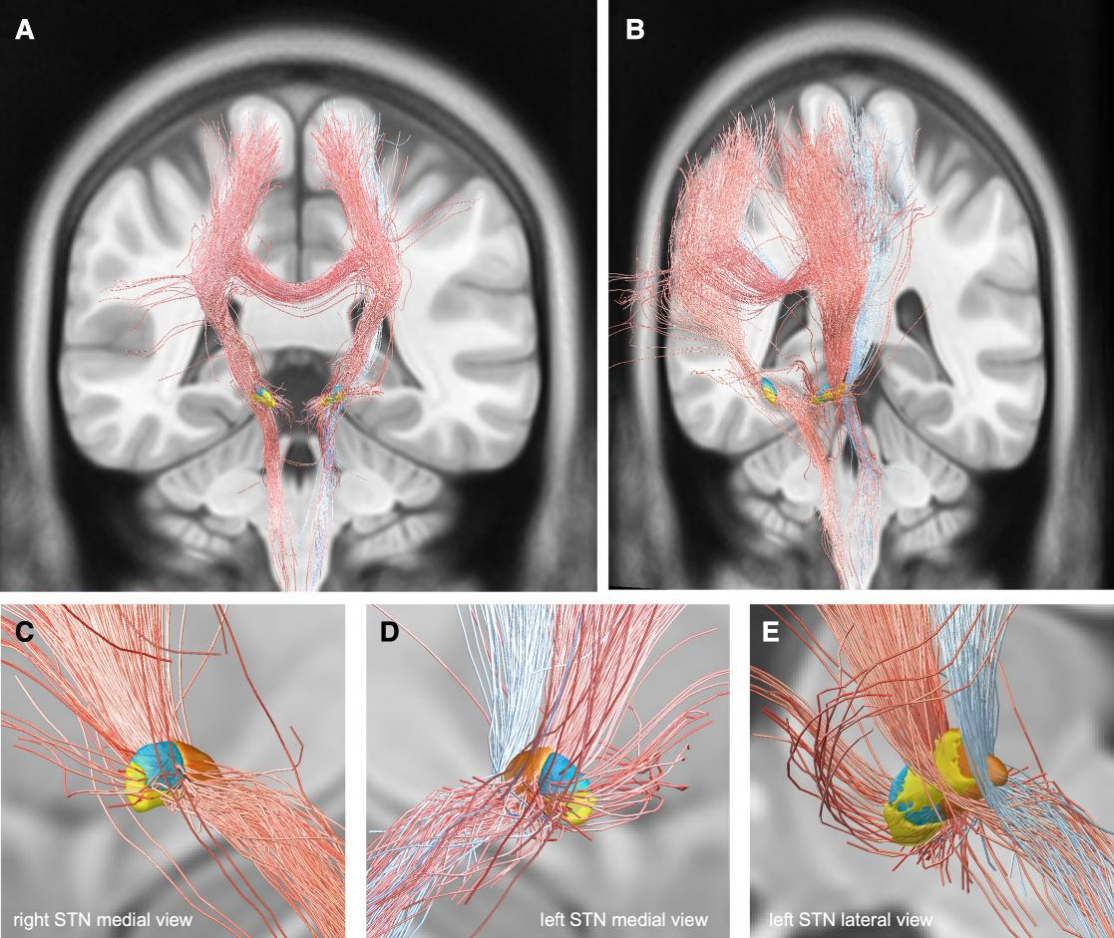
**Discriminative fibres connected with the volume of activated tissue (VAT) and associated with DBS-induced changes in antisaccade error rate.** Fifty percent of all significant fibres are shown coloured by their *T*-value (based on uncorrected *P* values < 0.05 in mass *t*-tests as described in the main text). Modulation of red fibres was associated with a DBS-induced increase of errors, while modulation of blue fibres was significantly associated with a DBS-induced reduction of errors. Motor subregion of the subthalamic nucleus (STN) in orange, non-motor subregion in cyan (associative) and yellow (limbic) taken from the DISTAL atlas. (**A**–**B**) Coronal and oblique views showing all significant discriminative fibres from bihemispheric VAT. (**C**) Close-ups of the right subthalamic nucleus in medial view. (**D**–**E**) Close-up of the left subthalamic nucleus in medial and lateral view. Most of the positively correlated fibres appear to enter the associative subregion of the STN, while the negatively correlated fibres enter more dorsally.

**Figure 6:**
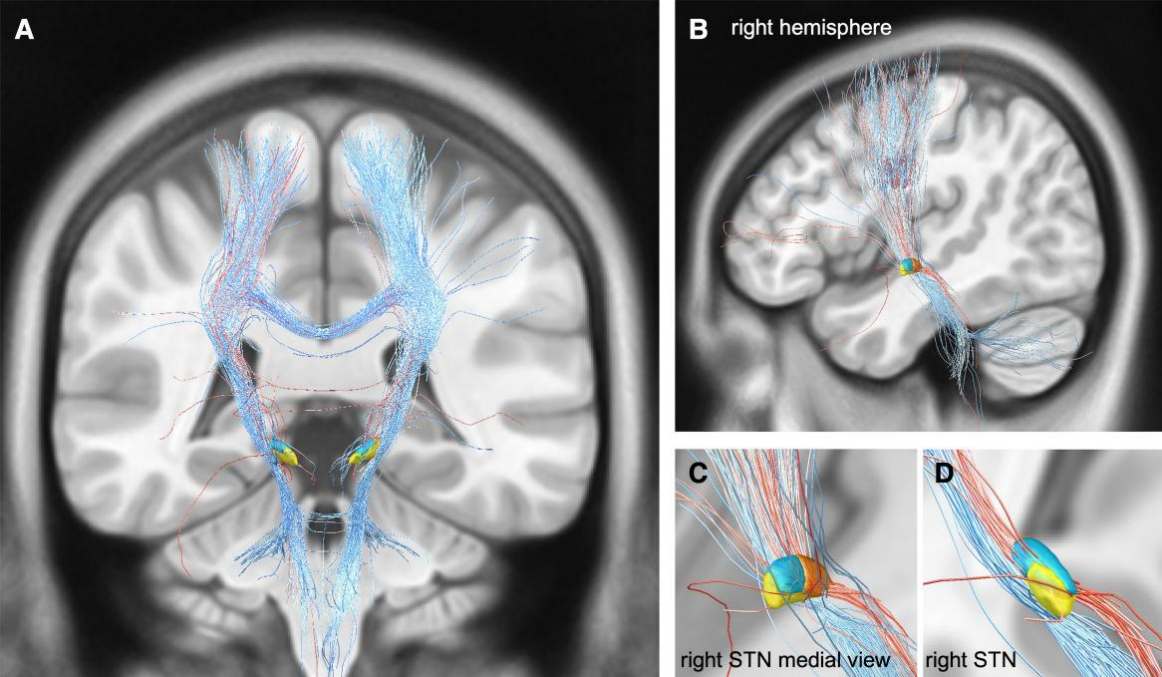
**Discriminative fibres connected with the volume of activated tissue (VAT) and associated with DBS-induced changes in antisaccade latency.** Only significant fibres are shown coloured by their *T*-value (based on uncorrected *P* values < 0.05 in mass *t*-tests as described in the main text). Red tracts were positively, blue tracts negatively correlated with change scores, i.e. modulation of red fibres was associated with a DBS-induced latency increase, and modulation of blue fibres was associated with a DBS-induced latency decrease. Motor subregion of the subthalamic nucleus (STN) in orange, non-motor subregion in cyan (associative) and yellow (limbic) taken from the DISTAL atlas. (**A**) Coronal view from posterior showing all significant discriminative fibres from bihemispheric VAT. (**B**) Lateral view on the fibres connected with the right VAT. (**C**–**D**) Close-ups of the right subthalamic nucleus. Positively and negatively correlated fibres seem to be distinct tracts with red fibres entering the dorsal STN, and blue fibres passing by laterally.

The whole-brain connectivity maps based on voxel-wise Spearman correlations are displayed in Fig. 3. The whole-brain fibre connectivity profiles are shown for antisaccade error rate in Fig. 5, respectively, for antisaccade latency in Fig. 6.

In the leave-one-out validation, the DBS effect of single individuals on error rate (*R* = 0.470, *P* = 0.046) but not latency (*R* = −0.09, *P* = 0.375) was predictable by linear models trained on the respective discriminative fibre profiles of the remaining 13 participants. That means that higher similarity of the individual set of discriminative fibres to the group fibre profile correlated with a larger DBS-induced effect on the antisaccade error rate.

Most fibres associated with a larger DBS-induced increase in antisaccade error rate entered the ventromedial portion of STN and projected onto the prefrontal cortex encompassing FEF, SEF and neighbouring areas bilaterally (Fig. 5A–C). In contrast, a smaller proportion of fibres that was associated with a DBS-induced decrease in antisaccade error rate entered the left dorsolateral portion of the STN and projected more dorsally onto the left prefrontal and motor cortex (Fig. 5A–C).

Regarding the discriminative fibres for DBS-induced antisaccade latency changes, it became clear that fibre tracts positively or negatively correlated with the change in antisaccade latency differed in their anatomic relation to the STN (Fig. 6). Fibres associated with a DBS-induced decrease in antisaccade latency passed by the STN at its lateral border and projected via the internal capsule onto the superior frontal and pre-central gyrus and downstream to the brainstem and cerebellum potentially consistent with corticotectal, -bulbar and -cerebellar tracts originating from the frontal eye fields and sensorimotor cortex. In a *post hoc* analysis, the proportion of the VAT localized outside the STN was not predictive for the change in antisaccade latency (*ρ* = −0.481, *P* = 0.081), suggesting that the effect was specific for the laterally passing fibres rather than generated by unspecific stimulation of non-STN tissue. Connectivity of the VAT with a smaller proportion of fibres located medial from the first fibre tract was associated with an increase in antisaccade latency with DBS instead. These fibres projected onto the same cortical areas but, in contrast to the first tract, appeared to enter the motor and associative subregions of the STN (Fig. 6).

## Discussion

### Summary of findings

In this study, we explored how the exact location of the volume of activated tissue and its structural connectivity affect response inhibition in the antisaccade task in persons with Parkinson’s disease treated with DBS in the STN. First, we showed that the DBS effect on antisaccade error rates hinged on the proportion of VAT intersection with the non-motor subregion of the STN and on structural connectivity between the VAT and several prefrontal regions involved in oculomotor control. Further, antisaccade latency tended to decrease in most participants when STN-DBS was switched on. This effect was larger when a ‘smaller’ portion of the VAT intersected with the motor subregion of the STN, lending credence to the idea that faster initiation of voluntary saccades observed with STN-DBS may relate to stimulation of adjacent structures outside the STN. This interpretation was supported by the identification of two distinct fibre tracts, one traversing the STN, the other one passing the STN laterally, which were associated with differential effects on latency changes.

### DBS of non-motor STN regions impairs selective response inhibition

In line with most previous studies,^23,50,51^ DBS induced no consistent effect on the rate of antisaccade errors on a group level. According to our data, the detrimental effect on response inhibition observable in some patients with STN-DBS might originate from rather ventromedial locations of the VAT within the STN. Another possible source of disinhibition might be the modulation of connections between the VAT and cortical oculomotor control regions such as the FEF and ACC which are involved in the inhibition of reflexive saccades towards the visual target.^42,52,53^

Comparable studies investigating the impact of VAT locations on response inhibition are scarce. A previous study exploring effects of DBS amplitude on antisaccades reported that a higher overlap between the VAT and the entire STN was associated with lower antisaccade error rates in the DBS-on state. Further, location of the active DBS contacts outside of the STN was associated with detrimental effects on the error rate.^54^ Generalizability may, however, be hampered by small sample sizes for VAT reconstructions, lack of consideration of behavioural changes from DBS-off state and by the fact that subregions within the STN were not differentiated.

Our results may also link to previous reports of activations of different contacts of the same DBS leads on response inhibition. For instance, unilateral stimulation of the most ventral contact impaired performance in a GO/No-go task compared with dorsal stimulation.^55^ Conversely, dorsal stimulation improved selective inhibition of conflicting impulses in a Simon task.^56^ As such, our findings are in line with these studies supporting deteriorated performance in response inhibition tasks with stimulation in the non-motor subregion of the STN. Conversely, the whole-brain connectivity profile suggests that stimulation of the left motor subregion of the STN may be associated with improved inhibition of antisaccade errors. Given that neurons in the STN activated during saccades tend to be located medial from those involved in manual responses^57^ and connections of STN with FEF and SEF are situated medial to those connecting the STN with the pre-SMA,^13^ it remains to be determined whether our results are directly transferable to manual response inhibition tasks.

Of note, effects of VAT location may vary in tasks requiring a general stopping of all ongoing actions instead of selective inhibition of a specific reflexive response to allow selection and execution of a voluntary response. For instance, stimulation via ventrally located contacts led to faster stopping times in a stop-signal task.^58^ On the same note, Lofredi and colleagues showed that significantly increased stopping times induced by STN-DBS were linked to connectivity of the VAT with the right pre-SMA and the inferior frontal gyrus.^59^ The opposing results from paradigms assessing global versus selective inhibition capacities support the conceptual and behavioural dissociation of two distinct forms of response inhibition.^60^

### Improved antisaccade latency may be an off-target effect

A decreasing effect of STN-DBS on antisaccade latency has been to date the most consistent finding.^24,50,61-63^ Of note, decreased response times (in absence of a simultaneous increase of errors) are equivalent to a relative normalization considering that antisaccade latency is typically increased in Parkinson’s disease compared with healthy individuals.^24^ Visualization of connected fibre tracts revealed that stimulation of fibre tracts passing by the STN laterally predicted latency decrease, while current delivered at fibres traversing the STN caused an opposite effect. It can thus be suggested that DBS-induced latency reduction may be rather driven by modulation of cortical projections bypassing the STN than an effect of modulating neuronal activity in the STN itself. A similar effect has been noted for DBS of the anterior limb of the internal capsule in individuals treated for depression or obsessive-compulsive disorder.^51^ Here, stimulation of fibres of the anterior internal capsule enhanced cognitive control capacity supporting the hypothesis that DBS effects may be exerted via long-range modulation of cortical projections.^51^

The clear distinction of negatively and positively correlated discriminative fibres on the level of the STN disappeared at the cortex level as the fibres tended to convergently project onto the same frontal regions. Corticotectal and corticopontine tracts connecting the prefrontal oculomotor regions with the superior colliculus and brainstem gaze control centres are located close to the lateral antero-superior border of the STN.^64^ Modulation of these fibres via current spread may facilitate the generation of voluntary saccades without causing the sustained lateral fixation observed as a side effect with ill-positioned leads or high stimulation amplitudes.^65^ Besides these direct projections, input from the cortical oculomotor control regions reaches the superior colliculus via the direct, indirect and hyperdirect basal ganglia pathways.^66^ As such, it seems that the DBS effect is dependent on whether the fibres are involved in basal ganglia pathways or directly project onto down-stream gaze control regions.

### Limitations

First, the parcellation of the STN into its subregions has been achieved using an atlas-based approach which does not account for any individual anatomical deviations. Furthermore, the functional division of the STN into two distinct region poses an oversimplification of the actual connectivity patterns emerging between frontal cortices and the STN. While there is indeed a dorsolateral-ventromedial gradient from preferentially motor to cognitive and limbic inputs, convergence of different cortical projections within the entire STN is large and without strict borders between motor and non-motor subregions.^13,67^ Still, the subdivision into distinct regions has been proven to be a useful model of the actual anatomical conditions when studying DBS effects.^12,18^

Secondly, the stimulation settings of some subjects had been optimized using imaging-guided approaches.^68^ Accordingly, the VAT were predominantly localized within the motor subregion of the STN. Given potential relationships between saccade alterations and stimulation of structures outside the STN as suggested by our results, further investigations in patients with suboptimal lead or VAT locations may, in fact, be helpful to confirm our findings.

Thirdly, we cannot account for individual differences in structural connectivity between participants using a normative connectome. Relying on the assumption of similar connectivity profiles across participants is a clear limitation, although this approach has been used and validated in studies on DBS effects before.^47,59^ Normative connectomes are based averaged high-quality data sets of large numbers of participants, resulting in high signal-to-noise levels. Furthermore, lacking preoperative patient-specific connectivity data, a connectivity analysis would have otherwise been impossible in this study. While the subjects included in the PPMI dataset did not differ from our cohort regarding age or sex, disease stage might have been more advanced in our participants since PPMI aimed towards recruitment of early-stage Parkinson’s disease.^45^

The sample size is small, and generalizability of our results may be limited. Since DBS-off and DBS-on conditions were recorded on the same day with a relatively short wash-out period in between, carry-over effects into the DBS-off condition cannot be completely excluded.

All participants were recorded after withdrawal of their regular dose of dopamine replacement therapy which might be considered a strength of the study since it excludes effects of dopaminergic medication on the results to a large extent (long lasting effects > 12 h cannot be entirely excluded). On the other hand, previous evidence suggests differential effects of DBS on antisaccade performance dependent on medication state {Citation}. Hence, our findings may be limited to the ‘off’ medication state.

## Conclusions and clinical implications

We confirmed previous studies^18,19^ that stimulation of the non-motor, i.e. associative and limbic subregions of the STN can deter cognitive processes, here response inhibition. As discussed above, this may apply particularly in scenarios in which selective inhibition of one reflexive response is required to allow the selection of another voluntary action. In real life, we constantly apply selective inhibition so that Parkinson’s disease-patients with impulse control disorders may particularly suffer from their reduced ability.^69^ Additional impairment of response inhibition induced by STN-DBS may potentiate this tendency postoperatively, so that most centres include screening in for impulse control disorders in their preoperative screening. Our results lend credence to the idea that stimulation of associative-limbic subregions of the STN should be avoided, especially in patients with risk factors for impulse control disorders. With the increasing capacity of steering VAT with modern leads, actively avoiding a current spread into the ventromedial aspects of the (right) STN while retaining optimal control over motor symptoms may become feasible in more patients, even when leads are suboptimally located.

Our results regarding improved antisaccade latency with stimulation of a distinct set of fibres passing the STN laterally provide first preliminary evidence that STN-DBS of may have enhancing effects on voluntary eye movement control through modulation of prefrontal projections. If this finding is replicable in future studies on response inhibition, adapting DBS programs using patient-specific visualization of VAT and stimulated fibre tracts may not only be useful for avoiding side effects and increasing the therapeutic window but may even exert beneficial effects on cognitive control.

## Supplementary Material

fcad121_Supplementary_Data

## Abbreviations

ACC =: anterior cingulate cortex
DBS =: deep brain stimulation
DLPFC =: dorsolateral prefrontal cortex
FEF =: frontal eye field
MDS-UPDRS =: Movement Disorders Society revised Unified Parkinson’s Disease Rating Scale
MoCA =: Montreal Cognitive Assessment
PPMI =: Parkinson’s Progression Markers Initiative
ROI =: region of interest
SEF =: supplementary eye field
SMA =: supplementary motor area
STN =: nucleus subthalamicus
VAT =: volume of activated tissue
