# Supplementary material for: Stimulation of non-motor subthalamic nucleus impairs selective response inhibition via prefrontal connectivity
Source: Brain Commun. 2023 Apr 13;5(2):fcad121. doi: 10.1093/braincomms/fcad121 (PMC10128876; doi:10.1093/braincomms/fcad121)
Supplement: fcad121_Supplementary_Data [file fcad121_supplementary_data.docx]

**Supplementary Material**

**Supplementary Methods**

**Eye-tracking procedure**

The experiment took place in a sound-attenuated, darkened and electrically shielded room which the researcher monitoring the progress in an adjoining room. All participants were seated in an upright armchair with back support at distance of 70 cm from a computer monitor with a diagonal of 60 cm and with their head stabilized with chin and forehead rests.

An infrared video-based eye-tracker (EyeLink 1000 Plus, SR Research, Ontario, Canada) recorded positions of both eyes at a sampling rate of 500 Hz and an instrumental spatial resolution of 0.01°. The eye-tracker was calibrated and validated with a 9-point grid before each experimental block. The validation was repeated until average errors for all points were <1° compared to the result of the calibration. Moreover, to ensure precision within blocks, a drift correction prior to each trial was performed.

Each trial started with a red central fixation cue (diameter 1° visual angle) that was presented for 1000 ms in the middle of a black screen. It was followed by the appearance of a white lateral target stimulus located either 10° left or right from the initial fixation cue. The lateral stimulus was presented in equal numbers and random order to the left and right side of the screen. The lateral stimulus vanished after 1000 ms and was followed by a white central dot for drift correction and a subsequent interstimulus interval (blank black screen) that allowed participants to blink. The next trial started with a new red central fixation cue.

The participants were instructed to look at the exact opposite direction of the lateral stimulus as fast and precisely as possible as soon as it was presented on the screen. Ten practice trials were performed prior to the first antisaccade block of the experiment and verbal feedback was given to ensure that the participants understood the instructions. Practice trials were not recorded, nor analyzed. Between blocks, participants were given the opportunity to take breaks.

**Eye-tracking data processing and analysis**

The researcher who performed the analysis of the eye-tracking and EEG data sets (JW) was not involved in data collection and was blinded to the participants’ identities. A parsing system incorporated in the EyeLink 1000 software intersected the raw eye position data into visual events, i.e., saccades, fixations, and blinks. This event data set was analyzed in the statistical computing program R [75] using the Eyelinker package [34]. Peak velocity larger than 30°/s and a deflection > 0.1° were set as thresholds for saccade detection.

A directive error was defined as a saccade towards the lateral stimulus. Saccade latency was defined as the time from stimulus onset to the start of the first saccade regardless of whether the saccade was elicited in the correct direction. Trials were removed from further analysis when 1) the latency was in the anticipatory range (< 90 ms) or longer than two standard deviations from the individual mean latency of the participant, 2) the first saccade after stimulus onset had a starting position more than 3° lateral of central fixation dot, 3) a saccade with an amplitude smaller than 0.5 ̊ or larger than 15 ̊ was executed or 4) a blink occurred between stimulus presentation and the first saccade. The processing of the eye-tracking data led to the rejection of 16.1% ± 12.5% of trials in the DBS-off condition and 13.3% ± 10.9% in the DBS-on condition.

**Supplementary Tables**

| **subject** | **left STN** | | **right STN** | |
| --- | --- | --- | --- | --- |
|  | active contacts | amplitude (mA) | active contacts | amplitude (mA) |
| 1 | 5- (4%), 6- (18%), 7- (18%), 8- (60%) | 2.2 | 5- (26%), 6- (7%), 6- (7%), 8- (60%) | 2.2 |
| 2 | 1- (10%), 2- (84%), 3- (3%), 4- (3%) | 0.9 | 2- (54%), 4- (29%), 3- (17%) | 0.6 |
| 3 | 2- (10%), 3- (10%), 5- (10%), 6- (10%), 4- (30%), 7- (30%) | 1 | 2- (10%), 3-(20%), 4-(30%), 5-(20%), 6-(10%), 7-(10%) | 1.7 |
| 4 | 2- (30%), 5- (50%),6-(20%) | 2.3 | 2- (25%), 3 (15%) 4- (45%) | 2 |
| 5 | 2- (5%), 3- (3%), 4- (12%), 5- (22%), 6- (11%), 7- (47%) | 2 | 1- (10%), 2- (6%), 3- (24%), 4- (60%) | 3 |
| 6 | 2- (33%, 3- (33%), 4- (33%) | 1.7 | 2- (33%), 3- (33%), 4- (33%) | 1.2 |
| 7 | 3- (20%), 4- (20%), 5- (40%), 6- (20%) | 2.5 | 1- (5%), 2- (20%), 3- (20%), 4- (55%) | 1.2 |
| 8 | 3-(10%), 4-(10%), 5-(15%), 6-(40%), 7-(25%) | 2.7 | 3- (40%), 4-(40%), 6-(10%), 7-(10%) | 1.5 |
| 9 | 2- (45%), 3- (25%), 4- (9%), 5- (19%), 6- (5%) | 3.1 | 3- (5%), 4- (50%), 5- (40%), 6- (5%) | 3.5 |
| 10 | 1- (20%), 2- (28%), 3- (26%)-4(26%) | 0.5 | 5- (34%), 6- (33%), 7- (33%) | 1 |
| 11 | 5- (40%), 6- (30%), 7- (30%) | 2 | 2- (20%), 3- (40%), 4- (30%) | 1.8 |
| 12 | 2- (60%), 4- (15%), 7- (25%), | 1.5 | 3- (40%), 4- (40%), 5- (20%) | 1.5 |
| 13 | 1- (80%), 2- (20%) | 2.5 | 6- (100%) | 2 |
| 14 | 2- (5%), 5- (60%), 6- (35%) | 3.9 | 3- (25%), 5- (15%), 7- (30%), 8- (30%) | 2 |

**Supplementary Table 1: Individual DBS-programs of all participants.** Pulse width was kept at 60 ms and frequency at 130 Hz in all patients.

| **Brainnectome number** | **Brainnetome code** | **description** | **ROI** |
| --- | --- | --- | --- |
| SFG1 | A8m_L | left medial Brodmann BA (BA) 8 | SEF |
| SFG1 | A8m_R | right medial BA 8 | SEF |
| SFG5 | A6m_L | left medial BA 6 | SEF |
| SFG5 | A6m_R | right medial BA 6 | SEF |
| MFG1 | A9/46d_L | left dorsal BA 9/46 | DLPFC |
| MFG1 | A9/46d_R | right dorsal BA 9/46 | DLPFC |
| MFG3 | A46_L | left BA 46 | DLPFC |
| MFG3 | A46_R | right BA 46 | DLPFC |
| MFG4 | A9/46v_L | left ventral BA 9/46 | DLPFC |
| MFG4 | A9/46v_R | right ventral BA 9/46 | DLPFC |
| MFG6 | A6vl_L | left ventrolateral BA 6 | FEF |
| MFG6 | A6vl_R | right ventrolateral BA 6 | FEF |
| CG2 | A24rv_L | left rostroventral BA 24 | ACC |
| CG2 | A24rv_R | right rostroventral BA 24 | ACC |

**Supplementary Table 2:** Brainnetome regions of interests (ROI) used in this study.

|  | **age** | | **disease duration** | | **MoCA** | | **change in MDS-UPDRS III (Off-On)** | |
| --- | --- | --- | --- | --- | --- | --- | --- | --- |
|  | r | p | r | p | r | p | r | p |
| **change in antisaccade latency** | 0.11 | 0.7 | -0.20 | 0.6 | 0.21 | 0.5 | 0.17 | 0.7 |
| **change in antisaccade error rate** | 0.51 | 0.06 | 0.08 | 0.8 | 0.1 | 0.8 | -0.19 | 0.6 |
| **change in “regular” error rate (latency > 129 ms)** | 0.42 | 0.1 | 0.12 | 0.7 | -0.05 | 0.9 | -0.16 | 0.7 |
| **change in express error rate (89 ms < latency < 130 ms)** | 0.08 | 0.8 | 0.01 | >0.9 | 0.12 | 0.7 | 0.41 | 0.3 |

**Supplementary Table 3:** Spearman correlations between antisaccade measures and clinical features.
